# A descriptive study of health status and health related quality of life in selected outpatients with type 2 diabetes, pathological body mass index and cardiovascular risk in Spain

**DOI:** 10.1186/1758-5996-6-135

**Published:** 2014-12-06

**Authors:** Concepción Vidal-Peracho, Maria Orosia Lucha-López, Ana Carmen Lucha-López, José Miguel Tricás-Moreno, Elena Estébanez-De Miguel, Luis Bernués-Vázquez

**Affiliations:** Specialty Medical Center Grande Covián, SALUD, Avda. Alcalde Caballero, 196, 50014 Zaragoza, Spain; Physiotherapy Research Unit, University of Zaragoza, C/Domingo Miral s/n, 50009 Zaragoza, Spain; Faculty of Health Sciences, Physiotherapy Research Unit, University of Zaragoza, C/Domingo Miral s/n, 50009 Zaragoza, Spain; Faculty of Health Sciences, University of Zaragoza, C/Domingo Miral s/n, 50009 Zaragoza, Spain

**Keywords:** Diabetes mellitus, Type 2, Obesity, Cardiovascular risk, Health related quality of life, Health status

## Abstract

**Background:**

Ottawa Charter defined health as a resource for everyday life and as an important dimension of health related quality of life (HRqol). Diabetes and obesity have repeatedly been shown as diseases that diminish health status and HRqol. The aim of this study was to measure health status and HRqol in a Spanish sample of obese patients with type 2 diabetes at cardiovascular risk and analyze behavioural, biological and social determinants of health.

**Methods:**

Outpatients from external specialized clinic in Endocrinology were evaluated. Measurements: sex, age, family history, employment status, comorbidities, pain, lifestyle habits, anthropometrics, blood pressure, blood analysis and HRqol with COOP/WONCA questionnaire (7 dimensions). Statistics: univariate, bivariate, multivariate and comparative analysis.

**Results:**

Mean age was 59.1 ± 7.6 [95%IC: 56.6-61.6], 74% were women and 63.2% were physically active. WONCA values were; summary index (SI): 18.7 ± 4 [95%IC: 17.3-20] (maximum 35); physical fitness: 3.3 ± 1, feelings: 2.3 ± 1.1, social activities: 1.5 ± 1, daily activities: 2.1 ± 1.2, change in health: 2.7 ± 0.9, overall health: 3.6 ± 0.7 and pain: 3.5 ± 1.2 (maximum 5). High fibrinogen values (339.3 ± 85.8 [95%IC: 309.8-368.8]) negatively influenced pain visual analogic scale (p = 0.029). Physically active patients (63.2%) had better values in daily activities dimension (p = 0.025). More than the half of the sample (51.5%) reported a good quality of sleep, but the pain worsened it (p = 0.040). High BMI values (34.8 ± 5.8 [95%IC: 32.9-36.7]) harmed the COOP-WONCA SI (p = 0.009). High glycated hemoglobin (HbA1c) values (6.8 ± 1.3 [95%IC: 6.3-7.2]) had a negative impact on COOP-WONCA SI (p = 0.018). Nor tailored diet (15.8%) or being employed (18.4%) influenced the HRqol. The regression that best models COOP-WONCA SI was adjusted for BMI and HbA1c. SI = 3.509 + 0.335BMI +0.330HbA1c.

**Conclusions:**

HRqol was worse than in general population, but better than in previous studies of diabetes patients, without differences by sex or age, though feelings, daily activities and pain dimensions scored worse than in these studies.

Higher levels of HbA1c, obesity and procoagulative state had a negative impact in these last dimensions. Pain impaired quality of sleep and physical activity had a positive impact in daily activities. BMI and HbA1c modeled the HRqol.

## Background

Since it was defined by the WHO in 1966 as “individuals’ perception of their position in life in the context of the culture and value systems in which they live and in relation to their goals, expectations, standards and concerns” [1], until today, health related quality of life (HRqol) is an index used increasingly in clinical practice because it provides additional information to the traditional clinical measures of the impact of disease and treatment outcomes in the welfare of people, considering the complexity of their biopsychosocial environment.

The Ottawa Charter [2] defined health as a resource for everyday life. Good health is a major resource for personal development and an important dimension of HRqol.

Diabetes and obesity have repeatedly shown as diseases that diminish health status and HRqol due to the functional consequences that entail, the changes in lifestyle associated with their treatment and the comorbidities and complications that often accompany them [3–5].

A better understanding of the factors affecting health status and HRqol of diabetic obese patients allows more accuracy in the modification of environment and lifestyle, and avoids subsequent disease complications [6].

Health measurement must contain elements of physical, mental, and social life in order to programme health promotion beyond healthy lifestyles that should be adapted to the individual and local needs [2].

Therefore, the aim of this study was to measure health status and HRqol in a Spanish sample of patients with pathological body mass index (BMI), type 2 diabetes and cardiovascular risk and analyze behavioural, biological and social determinants of health that influenced their health status and HRqol.

## Methods

### Study and sample selection

A nonexperimental descriptive research was performed, with a ex post facto, correlational, comparative and cross-sectional study, in a sample of 38 caucasian people with type 2 diabetes, pathological BMI and cardiovascular risk who attended specialized Endocrinology and Nutrition outpatient clinic at the Hospital Royo Villanova (Grande Covian Specialty Medical Center) in Zaragoza, Spain.

Inclusion criteria were: diagnosis of type 2 diabetes, according to criteria of the American Diabetes Association (ADA) [7], body mass index (BMI) greater than or equal to 25 and being older than 45 years.

All patients who met the inclusion criteria, attending the consultation were included. They voluntarily accepted participation after receiving the right information, so we made a convenience, consecutive, non-probabilistic sample method. All signed an informed consent and they were told that they could leave the study at any time and for any reason. The Department of Physical Therapy and Nursing in University of Zaragoza authorized the study, which complied with the ethical requirements of the Declaration of Helsinki [8].

### Measurements

The following data were collected:

Anamnesis: sex, age, age at the onset of diabetes, family history, employment status, comorbidities, and lifestyle related factors as dietary habits (type of diet in the past year), hours and intensity of physical activity and smoking habits.

Pain visual analog scale (PAIN VAS) (the horizontal form) [9]. Pain was characterized according to its severity using this numeric scale: 0–1.9; absence of pain, 2–4.9; light pain, 5–6.9; moderate pain, 7–9.9; intense pain, 10; worst pain imaginable [10].

Sleep Quality: the objective was to evaluate the deterioration of the quality of sleep. Patients were asked to rate their quality of sleep in one of the following three categories: good - fair - poor [11].

Blood pressure: measurement of systolic blood pressure (SBP) and diastolic blood pressure (DBP) was performed with a sleeve adapted to obese patients, after a period of about fifteen minutes in a sitting position, with one tube, clock tensiometer (Riester Corporation).

Anthropometrics: all data were obtained according to the protocol of the International Society for the Advancement of Kinanthropometry (ISAK) [12]. Height was measured with wall stadiometer (Seca-Health Line: removable stadiometer, scope 30–220 cm), weight with electronic scale and fat percentage with bioelectrical impedance analysis (Biological Company: TANITA TBF 300) and waist circumference with inextensible tape (TECSYMP Instruments: plastic tape, measure 0–2 m). Body mass index (BMI) was calculated as weight in kilograms divided by the square of the height in meters.

Based on body fat percentage, obese subjects were defined as those with fat percentages above normal values, which are 12 to 20% in adult men and 20 to 30% in adult women [13].

Related to body mass index participants were classified with adult gradation of Spanish Society for Obesity Study, Sociedad Española para el Estudio de la Obesidad (SEEDO); overweight grade I: 25,0-26,9; overweight grade II: 27,0-29,9; obesity grade I: 30,0-34,9; obesity grade II: 35,0-39,9; obesity grade III: 40,0-49,9; obesity grade IV ≥ 50 [14].

Blood analysis: the assessment of blood parameters was performed in the laboratory of Grande Covian Specialty Medical Center, by drawing blood, after a fasting period of not less than 12 hours. The following parameters were analyzed.

Total cholesterol, (HDL)-cholesterol, (LDL)-cholesterol, triglycerides and fibrinogen were determined by nephelometry (DADEBehring Corporation). Glucose and glycated hemoglobin (HbA1c) were determined using a selective modular analyzer (Roche Corporation). Accreditation National Entity (ENAC) validated the quality control of the laboratory (accreditation number: 742/LE1586).

HRqol: it was measured with the generic questionnaire developed by a group of doctors of the World Organization of National Colleges, Academies and Academic Associations of General Practitioners/Family Physicians (WONCA) in the Darmouth Primary Care Cooperative Information Project (COOP Project) (Hanover, New Hampsshire EE.UU): Functional Health Assessment Charts: COOP/WONCA charts. This questionnaire has been validated in Spanish population [15] and it has been widely used in diabetic population [5] because it has the next characteristics: person centred (somatic, psychological and social profile), clinical relevance, reliability and validity, sensitive to moderate changes in health, in the same way than traditional tests for the evaluation of the HRqol such as 36-Item Short Form Health Survey (SF-36) [15]. On the other hand, it is easier to use, easy to assess and generally usable and acceptable.

The COOP/WONCA 7-item/charts questionnaire was chosen. The seven items measure the next HRqol domains: physical fitness, feelings, social activities, daily activities, change in health, overall health and pain. Each of the charts possesses one question referring to events that have happened in the last two weeks and each of the questions has 5 possible answers. The scale is a 5-point ordinal scale (1–2 - 3–4 - 5, the higher the score, the worse the perception of HRqol). It is possible to add the results from each of the domains and provide a COOP/WONCA summary index (SI), whose range is between 7 and 35 points, but it has not been established a cut off of normality.

The seven questions are:

Physical fitness

During the past 2 weeks…What was the hardest physical activity you could do for at least 2 minutes?

1. Very heavy, (for example) run, at a fast pace
2. Heavy, (for example) jog, at a slow pace
3. Moderate, (for example) walk, at a fast pace
4. Light, (for example) walk, at a medium pace
5. Very light, (for example) walk, at a slow pace or not able to walk

Feelings

During the past 2 weeks…How much have you been bothered by emotional problems such as feeling anxious, depressed, irritable or downhearted and sad?

1. Not at all
2. Slightly
3. Moderately
4. Quite a bit
5. Extremely

Social activities

During the past 2 weeks…Has your physical an emotional health limited your social acivities with family, friends, neigbours or groups?

1. Not at all
2. Slightly
3. Moderately
4. Quite a bit
5. Extremely

Daily activities

During the past 2 weeks…How much difficulty have you had doing your usual activities or tasks, both inside and outside the house because of your physical and emotional health?

1. No difficulty at all
2. A little bit of difficulty
3. Some difficulty
4. Much difficulty
5. Could not do

Change in health

How would you rate your overall health now compared to 2 weeks ago?

1. Much better
2. A little better
3. About the same
4. A little worse
5. Much worse

Overall health

During the past 2 weeks…How would you rate your health in general?

1. Excellent
2. Very good
3. Good
4. Fair
5. Poor

Pain

During the past 2 weeks…How much bodily pain have you generally had?

1. No pain
2. Very mild pain
3. Mild pain
4. Moderate pain
5. Severe pain

A conceptual form of the instrument can be found at the following link: http://www.dartmouthcoopproject.org/coopcharts_overview.html.

### Statistical analysis

Data were analyzed with SPSS version 16.0 and the following calculations were performed:

Univariate analysis: the mean, standard deviation (SD) and 95% confidence interval (CI) for quantitative variables and percentages for ordinal and qualitative variables.

Bivariate analysis: we conducted the study on the magnitude of the association between quantitative variables with correlation coefficient rho of Spearman, between sociodemographic and clinical factors and COOP-WONCA responses.

Multivariate analysis: multivariate linear regression to model the relationship between the dependent variable COOP/WONCA summary index and the socio-demographic and clinical factors as explanatory variables. The assumptions of the model have been checked: linearity, homoscedasticity, uncorrelatedness, normality.

Comparative study, for comparison of hypothesis in independent samples:

- Two independent samples: Mann Whitney *U* test was applied. Independent variables considered were: sex (men/women), employment status (employed/not employed or retired), dietary habits (not diet/diet adapted to the clinical situation), physical activity (not physical activity / physical activity), HbA1c levels: below the set target of 7% (yes/no).
- More than two independent samples: Kruskall Wallis test was applied. Independent variables considered were: pain severity, BMI grades and sleep quality (good, fair and poor).

A level of significance of 5% (p <0.05) was established to reject the null hypothesis.

## Results

Description of the general determinants of health of the sample, such as age, age at the onset of diabetes, employment status, presence of comorbidities, and lifestyle, can be found in Table 1. It can be seen that the mean duration of diabetes in the sample was more than 10 years.

**Table 1:** **General determinants of health of the sample**

Women	74% (n = 28)
Men	26% (n = 10)
Age (years)	59.1 ± 7.6 [56.6-61.6]
Mean age at the onset of diabetes (years)	48.7 ± 10.2 [45.4-52.1]
Employed	18.4% (n = 7)
Percentage of the subjects with some musculoskeletal disorder	97.4% (n = 37)
Percentage of the subjects physically active	63,2% (n = 24)
Percentage of the subjects with adapted diet in the last 12 months	15.8% (n = 6)
Smoking habits	0% (n = 0)
PAIN VAS	6.5 ± 2.5 [5.7-7.3]

Mean ± SD and [95% CI] for quantitative variables and percentages for qualitative variables.

Response rates to the questions of the HRqol questionnaire COOP-WONCA, mean score, standard deviation and 95% confidence interval for each of the dimensions and summary index of the questionnaire are described in Table 2.

**Table 2:** **Descriptive analysis of the responses of the COOP-WONCA**

Question	Response	Percentage (n = 38)	Mean ± SD [95% CI]
Physical fitness	Very heavy	10.5	3.3 ± 1 [2.9-3.6]
	Heavy	2.6	
	Moderate	39.5	
	Light	44.7	
	Very light	2.6	
Feelings	Not at all	28.9	2.3 ± 1.1 [2–2.6]
	Slightly	31.6	
	Moderately	18.4	
	Quite a bit	21.1	
	Extremely	0	
Social activities	Not at all	73	1.5 ± 1 [1.2-1.9]
	Slightly	16.2	
	Moderately	0	
	Quite a bit	8.1	
	Extremely	2.7	
Daily activities	No difficulty at all	44.7	2.1 ± 1.2 [1.8-2.6]
	A little bit of difficulty	13.2	
	Some difficulty	26.3	
	Much difficulty	15.8	
	Could not do	0	
Change in health	Much better	16.2	2.7 ± 0.9 [2.3-3]
	A little better	13.5	
	About the same	62.2	
	A little worse	5.4	
	Much worse	2.7	
Overall health	Excellent	0	3.6 ± 0.7 [3.4-3.9]
	Very good	2.7	
	Good	45.9	
	Fair	40.5	
	Poor	10.8	
Pain	No pain	10.8	3.5 ± 1.2 [3–3.9]
	Very mild pain	8.1	
	Mild pain	21.6	
	Moderate pain	40.5	
	Severe pain	18.9	
Summary index			18.7 ± 4 [17.3-20]

Clinical health status, including blood pressure, anthropometrics and blood analysis is shown in Table 3, in which the reference values, and the percentage of the sample in these values are included.

**Table 3:** **Clinical health status of the sample**

	Values in the sample Mean ± SD [95% CI]	Reference values	Percentage values of the sample within reference
SBP (mmHg)	133.6 ± 13.8 [128.4-138.9]	< 140*	47.4% (n = 18)
DBP (mmHg)	79.3 ± 5.9 [77–81.6]	< 90*	68.4% (n = 26)
BMI	34.8 ± 5.8 [32.9-36.7]	18.5-24.9*	0% (n = 0)
Weight (kg)	85.5 ± 14.9 [80.6-90.4]		
Waist circumference (cm)	106.7 ± 12.2 [102.7-110.7]	< 88 women/102 men**	5.3% (n = 2) women/13.2% (n = 5) men
Fat percentage	38.8 ± 7.6 [36.3-41.3]	10-20 men/20–30 women*	0% (n = 0)
Total cholesterol (mg/dl)	171.9 ± 23.4 [164–180]	< 200*	91.7% (n = 33)
HDL-c (mg/dl)	56.8 ± 11 [53–60.5]	> 50*	66.7% (n = 24)
LDL-c (mg/dl)	92.1 ± 20.7 [85.2-99.1]	< 130*	89.5% (n = 34)
Triglycerides (mg/dl)	123.1 ± 76.7 [97.2-149]	< 200*	91.7% (n = 33)
Glucose (mg/dl)	141.3 ± 40.7 [127.5-155]	< 100***	11.1% (n = 4)
HbA1c (%)	6.8 ± 1.3 [6.3-7.2]	< 5.7% reference/7%* target**	23.7% (n = 9) reference/44.7% (n = 17) target
Fibrinogen (mg/dl)	339.3 ± 85.8 [309.8-368.8]	200-400****	77.1% (n = 27)

^*^SEEDO 2007, ^**^ATP III 2002, ^***^ADA 2012, ^****^Gailani D, Neff AT 2008.

Drug prescription for different pathologies of the sample can be found in Table 4 and antidiabetic medications in Table 5.

**Table 4:** **Medications used**

Type of drug	Percentage of the sample
Drugs to control blood pressure	70.9% (n = 27)
Hypolipidemic drugs	87.1% (n = 33)
Oral antidiabetic and/or insulin	93.5% (n = 36)
Antiplatelet drugs	87% (n = 33)

**Table 5:** **Type of antidiabetic medications**

Type of antidiabetic drugs	Percentage of the sample
Oral antidiabetic + Insulin analogs: four doses	9,70%
Oral antidiabetic + Insulin analogs: three doses	25,80%
Oral antidiabetic + Insulin analogs: one dose	9,70%
Oral antidiabetic	41,90%
Insulin analogs: four doses	3,20%
Insulin analogs: three doses	3,20%

Table 6 shows the correlations with statistical significance, and the value of Spearman coefficient, between clinical factors and dimensions of HRqol and summary index of the COOP-WONCA questionnaire. More pain, worst values in anthropometric variables that quantify obesity and worst values in blood analysis variables that translate metabolic control are associated with poorer HRqol, especially in daily activities and overall health.

**Table 6:** **Correlations between clinical factors and the COOP-WONCA questionnaire**

	Physical fitness	Feelings	Daily activities	Overall health	Pain	WONCA Summary index
PAIN VAS					0.330*	
BMI			0.516**		0.387*	0.481**
Fat percentage			0.573**	0.364*	0.346*	0.522**
Weight (kg)			0.454**			0.375*
Waist circumference (cm)			0.511**			0.351*
HbA1c (%)	0.367*	0.338*	0.409*	0.503**		0.365*
Glucose (mg/dl)		0.432**	0.390*	0.347*		0.375*
Fibrinogen (mg/dl)			0.529**	0.396*		0.362*
Triglycerides (mg/dl)		0.342*				0.403*

**Correlation is significant at the 0.01 level (bilateral). *Correlation is significant at the 0.05 level (bilateral).

**Figure 1:**
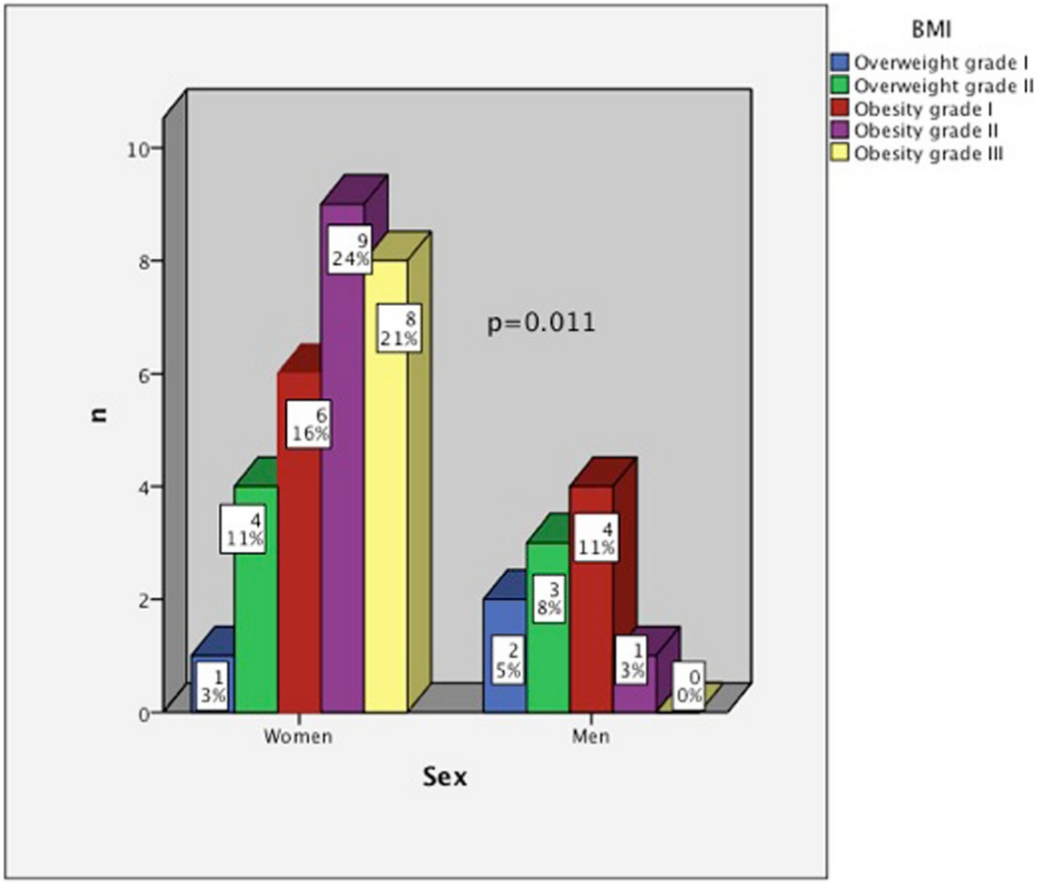
**Comparison of BMI between sexes.**

When grades of BMI by sex were compared with Mann Whitney *U* test, BMI in women was higher than in men, and while among women 21% (n = 8) had grade III obesity among men no case was collected (Figure 1).

**Figure 2:**
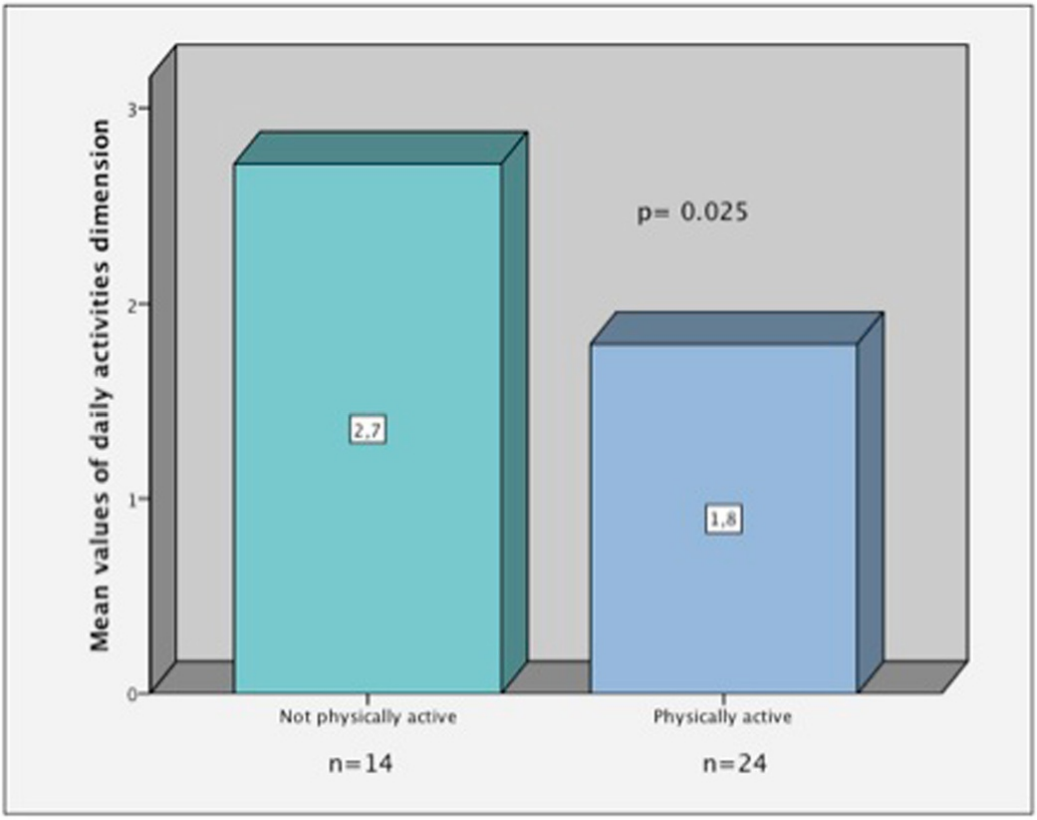
**Comparison of the mean values of the daily activities dimension of WONCA between active and non-active persons.**

**Figure 3:**
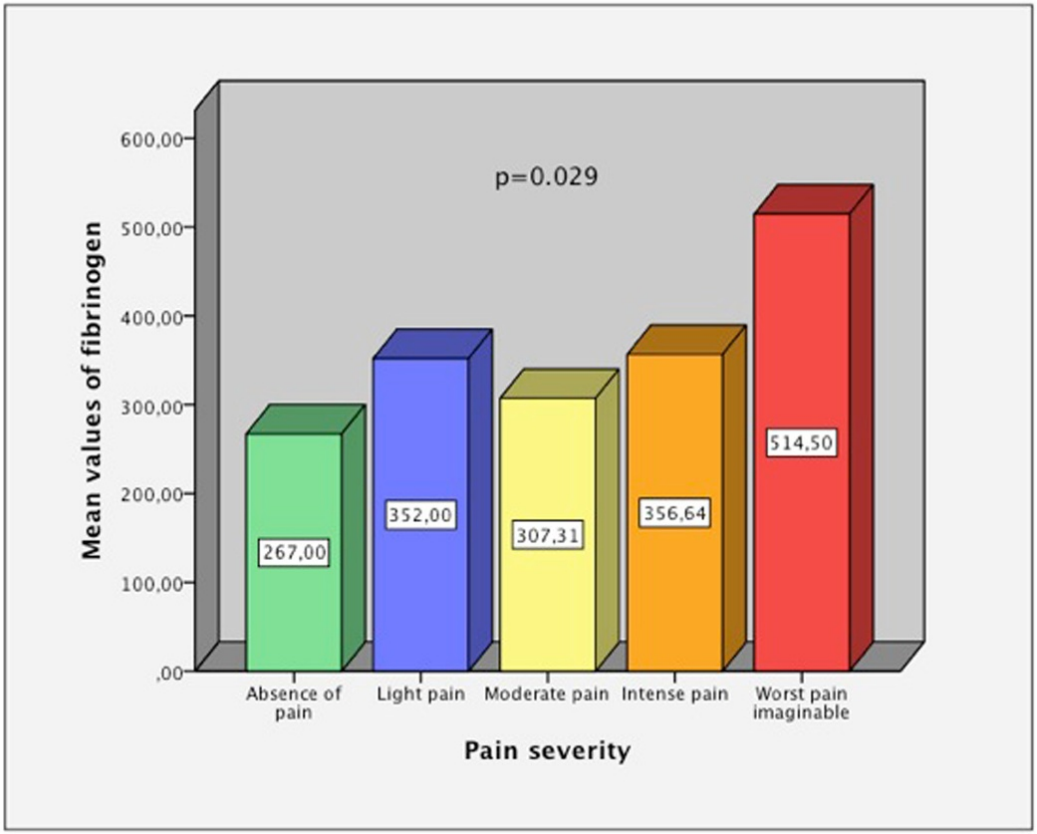
**Comparison of the mean values of fibrinogen according to pain severity.**

**Figure 4:**
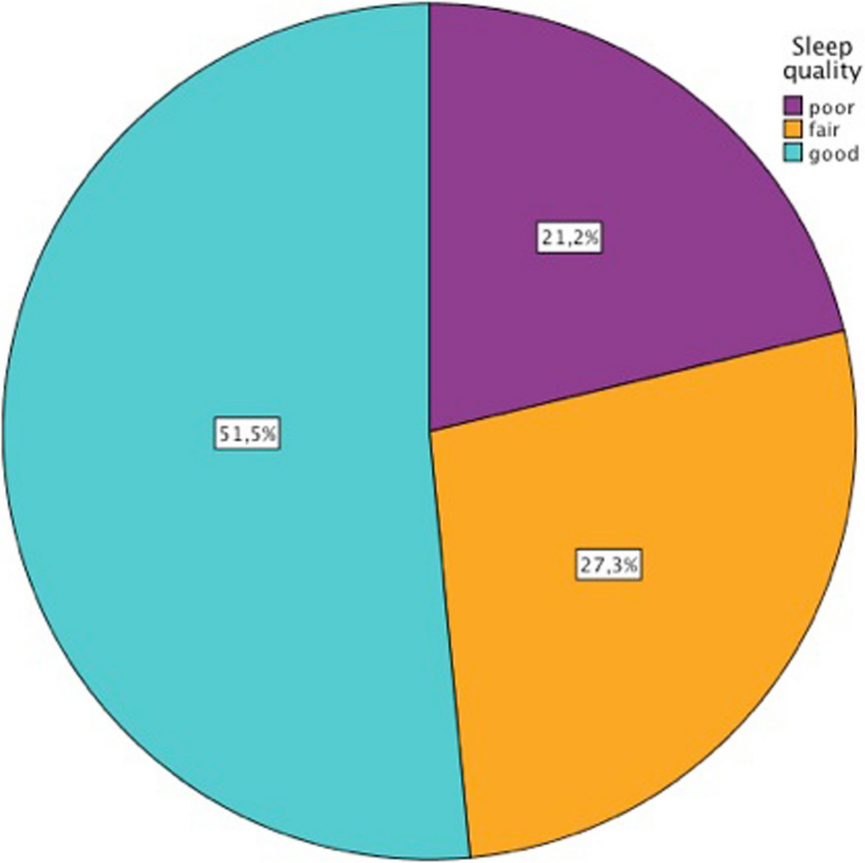
**Percentage of the sample in each of the categories of sleep quality.**

**Figure 5:**
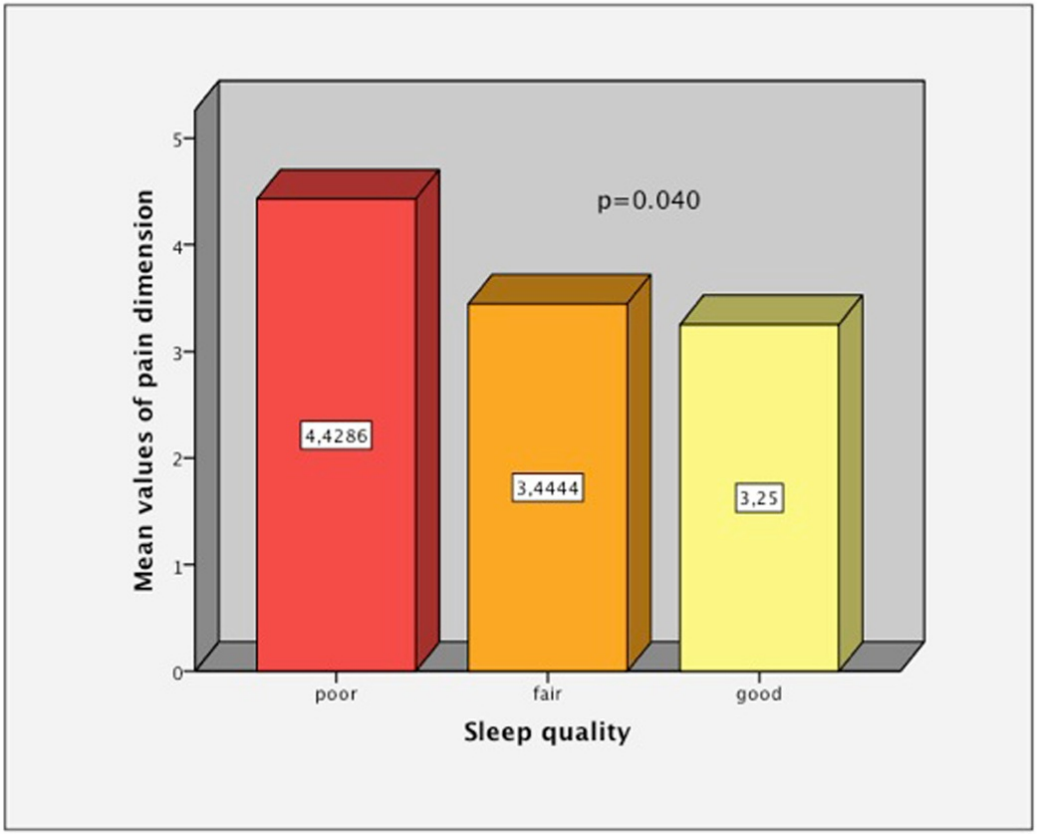
**Comparison of the mean values of pain dimension according to sleep quality.**

When the HRqol was compared between active people and those who were not, with Mann Whitney *U* test, a major difference was observed in the daily activities dimension (Figure 2), so people who were less active had lower HRqol in daily activities dimension than those who were more active.The comparative study with Kruskal-Wallis test, depending on the severity of pain, showed that there were statistically significant differences in fibrinogen values between the different levels of pain, with higher values when the pain was intense (Figure 3).The description of the quality of sleep of the sample (percentages of good, fair and poor sleep) can be viewed in Figure 4. When comparing, with Kruskal-Wallis test, HRqol depending on the sleep quality, it was found that people who slept worse scored higher on the pain domain of the COOP-WONCA (Figure 5).

Kruskall Wallis test, using grades of BMI as independent variable, shows significant differences in daily activities dimension and in the summary index of the questionnaire (Table 7). Therefore, most obese people have more difficulty performing daily activities and poorer HRqol in general.

**Table 7:** **Comparison of HRqol according to the degrees of IMC**

	Overweight grade I (n = 3)	Overweight grade II (n = 7)	Obesity grade I (n = 10)	Obesity grade II (n = 10)	Obesity grade III (n = 8)	p value
Daily activities	1 ± 0	1.1 ± 0.4	2.1 ± 1	2.6 ± 1	2.9 ± 1.4	0.012
COOP-WONCA Summary Index	16 ± 1.7	15.1 ± 1.9	18.2 ± 2.6	21.6 ± 4.5	19.6 ± 4.6	0.009

Mann Whitney *U* test, depending on the range of HbA1c, reveals significant differences in feelings, daily activities and overall health domains and in the summary index of the COOP-WONCA questionnaire (Table 8). Thus, a value of HbA1c above the target adversely affects the HRqol of our patients, especially the perception of feelings, daily activities and overall health.

**Table 8:** **Comparison of HRqol according to the values of HbA1c**

	HbA1c below the set target of 7% (n = 17)	HbA1c above the set target of 7% (n = 21)	p value
Feelings	1.9 ± 0.9	2.7 ± 1.2	0.042
Daily activities	1.7 ± 0.9	2.5 ± 1.3	0.036
Overall health	3.18 ± 0.5	4 ± 0.7	0.002
COOP-WONCA Summary index	16.8 ± 3	20.2 ± 4.3	0.018

Comparisons taking as independent variables employment status or diet habits in the last twelve months produced no statistically significant result in the variables of HRqol. So these variables have no influence on the quality of life in our sample.

The linear regression that best predict the value of the dependent variable summary index of COOP-WONCA involves the next explanatory variables BMI and HbA1c.

The equation, with all of the variables on the same scale, is the next:


The coefficient for BMI is 0.335. So for every unit increase in BMI, a 0.335 unit increase in summary index of COOP-WONCA is predicted, holding all other variables constant.

The coefficient for HbA1c is 0.330. So for every unit increase in HbA1c, a 0.330 unit increase in summary index of COOP-WONCA is predicted, holding all other variables constant.

The value of the Fisher-Snedecor F, for this model is 5.941, with a p value of 0.006. The F-statistic tests whether the overall regression model is a good fit for the data. The F value shows that BMI and HbA1c statistically significantly predict summary index of COOP-WONCA, so the regression model is a good fit of the data.

R2 value (also called the coefficient of determination) is 0.265, which is the proportion of variance in the summary index of COOP-WONCA that can be explained by BMI and HbA1c. You can see from our value of 0.265 that BMI and HbA1c explain 26.5% of the variability of summary index of COOP-WONCA.

## Discussion

This study showed that in our sample of outpatients with type 2 diabetes (mean glucose (mg/dl): 141.3 ± 40.7; mean HbA1c (%): 6.8 ± 1.3) and pathological BMI (mean: 34.8 ± 5.8), 74% were women, mean age was 59.1 ± 7.6, and mean age at the onset of diabetes 48.7 ± 10.2. 97.4% had some musculoskeletal disorder and punctuation in PAIN VAS was 6.5 ± 2.5. 63,2% were physically active and only 15.8% carried adapted diet in the last 12 months. They had acceptable metabolic control, facilitated by intensive medication use to control blood pressure, lipids, glucose and procoagulant state. Mean summary index of the COOP/WONCA was 18.7 ± 4 and physical fitness, overall health and pain were the dimensions of HRqol which scored worst.

The women in the sample had a higher degree of obesity, as in the study of Bosić-Zivanovic and colleagues [16]. No gender differences for any of the variables of HRqol were found, even though, other authors have identified the female as a factor associated with worse HRqol records [17].

The COOP-WONCA summary index questionnaire indicated a better HRqol than that newly obtained in a Spanish diabetic population [5] and worse than that of the general population [18], but specifically in the subscales of feelings, daily activities and pain, the mean scores were even worse than in the diabetic population already referred [5].

The biggest difference was observed in the pain subscale in which 40.5% of our sample reported moderate pain, severe pain 18.9% and only 10.8% no pain, which also differs from data obtained in 2009 in a diabetic population in our region [19] that reflected moderate pain only in 21.4% and severe pain in 3.1%, with a high percentage of patients with no pain 42.9%.

The high percentage of participants who had some musculoskeletal disorder may account for these results. The mean values in relation to the severity of pain were indicative of moderate pain and higher fibrinogen values negatively influenced records on this dimension. It is believed that high fibrinogen levels are related to situations of catabolic stress [20], which, in turn, could also have favoured the pain in our sample.

The average age of participants was 59.1 years, and despite its relative youth, a long average period of evolution of the disease over 10 years was observed. Unlike what was observed in the study by Esteban and Peña [5], age was not associated with any of the variables of HRqol, which may be because our research has used a more homogeneous group in middle and advanced age with no records in young people, as in the study by Jepsen et al. [3]. None of the study participants were smokers, which also differ from the 17.88% identified by Esteban and Peña [5].

The lack of employment activity did not influence any of the variables studied. The literature has not associated lack of employment activity with quality of life in diabetic patients; but it has been observed that unemployment worsens the quality of life in the general population, although this was not a direct result of health problems [21].

Only 15.8% of participants had made diet tailored to their illness in the past year. The diet change required to control the disease did not affect their HRqol, although previous studies have reported that changing eating habits was what most negatively affected the HRqol of the patients [4]. The dietary support they were given could have favoured dietary changes. They receive advice from the Doctor specializing in Endocrinology and Nutrition and also from the nurses working on the Specialty Service. Nurses carry out the work of counsellors and nutrition educators. They provide individualized advice and a series of generalized guidelines that are protocolized. Due to the low percentage of patients who performed adapted diet, after this study, an intensive nutrition education program that also included group education sessions was implemented.

63.2% of the sample performed physical activity, which positively influenced daily activities dimension of the questionnaire. Other authors have noted the association between the level of physical activity and perceived HRqol [3, 5], and it seems logical to observe the perceived benefits in daily activities.

More than the half of the sample (51.5%) reported a good quality of sleep, but the pain worsened it. We have not references where this relationship has previously been studied in diabetic patients, although it has been observed in the general population [22].

All patients in the sample had BMI and percentage of fat above the normal range, with a predominance of central distribution of fat. The high values of BMI and fat mass harmed the COOP-WONCA summary index, and daily activities and pain dimensions and worst values of BMI and fat mass are associated with poorer quality of life in these patients. Obesity as one of the comorbidities that affect the HRqol in diabetic patients had been previously identified in Spanish population of the urban area [5]. These results agree with those from Gough et al. [23], in which the negative impact and summatory effect of diabetes and obesity in the HRqol of British general population was also noted.

More than 10 years of diabetes evolution, obesity and procoagulant state [24] indicated that it was a group at high cardiovascular risk [25, 26].

Considering the criteria of American Diabetes Association (ADA) [27] and SEEDO [14], the percentage of patients within the recommended criteria almost equal to those found by Mengual and colleagues [28] (HbA1c: 54.8%, blood pressure: 29.6%, LDL: 40.6%) for glycosylated hemoglobin (HbA1c: 44.7%) and it is better for blood pressure (47.4%) and LDL (89.5%).

Despite the relatively good levels of glycosylated hemoglobin, higher values of this variable were associated with worse scores on all dimensions of WONCA, except for social activities and change in health, and they had a negative impact on feelings, daily activities, overall health and COOP-WONCA summary index. High HbA1c values have been previously associated with worse HRqol in the dimensions of physical and psychological health and social relationships [16].

The regression model with COOP-WONCA summary index as dependent variable confirmed the importance of BMI and HbA1c, because they were the clinical factors that predict the HRqol of our patients.

These findings have several limitations. First, the study includes possible selection bias, given the voluntary nature of participation. Second, due to strict inclusion criteria the size of the sample was quite limited, though data were sensible, as confidence intervals have shown. Third, the sample was biased in favour of females, which may have prevented finding differences in HRqol or health status between sexes. Fourth, because few studies have analyzed health in similar patients with type 2 diabetes, pathological body mass index and cardiovascular risk, future studies that confirm these findings are needed.

Despite this, future health promotion programs for similar samples could develop specific designs taking into account these outcomes. Intensive multidisciplinary educational programs (medicine, nursing, physiotherapy) may include content to help the patient in the management of diet, physical activity, blood glucose control and pain which would result in improved health status and HRqol.

## Conclusions

In summary, the HRqol of our patients with diabetes, pathological body mass index, and cardiovascular risk was worse than that of the general population, and better than previously recorded in diabetics, with no differences by sex or age, even though women had a greater degree of obesity and this factor has been identified as decisive in the perception of HRqol.

In the dimensions of feelings, daily activities, and especially in pain, worse scores than in other studies in diabetic patients were obtained. This scores in feelings and daily activities seemed influenced by levels of HbA1c, obesity also harmed daily activities.

The results of this study showed that predictors of HRqol were BMI and HbA1c, even though the acceptable diabetes control in a high percentage of the sample.

Physical activity benefited the performance of daily activities and pain impaired the quality of sleep.

## Authors’ information

The authors of this article are professors in the Faculty of Health Sciences, University of Zaragoza, center for teaching and multidisciplinary research in health sciences and members of the Physiotherapy Research Unit, state multidisciplinary research group in health sciences. This work meets one of the objectives of the group, that is the comprehensive analysis of the health of populations with special needs in order to meet the most crucial aspects of it and thus be able to program actions for health promotion taking into account the most relevant aspects for the population studied, from a multidisciplinary perspective.

## Abbreviations

ADA: American Diabetes Association
ATPIII: Adult treatment panel III
BMI: Body mass index
BP: Blood pressure
cm: Centimetre
COOP/WONCA: World Organization of National Colleges, Academies and Academic Associations of General Practitioners/Family Physicians (WONCA) in the Darmouth Primary Care Cooperative Information Project (COOP Project)
COOP/WONCA SI: COOP/WONCA summary index
DBP: Diastolic blood pressure
dl: Decilitre
ENAC: Accreditation National Entity
HbA1c: Glycated hemoglobin or glycosylated hemoglobin
HDL: High density lipoprotein
HRqol: Health related quality of life
ISAK: International Society for the Advancement of Kinanthropometry
kg: Kilogram or kilogramme
L: Litre
LDL: Low density lipoprotein
mg: Milligram
mmHg: Millimetre of mercury
SBP: Systolic blood pressure
SEEDO: Spanish society for the study of diabetes and obesity
PAIN VAS: Pain visual analogic scale.
